# Prioritizing Potentially Druggable Mutations with dGene: An Annotation Tool for Cancer Genome Sequencing Data

**DOI:** 10.1371/journal.pone.0067980

**Published:** 2013-06-27

**Authors:** Runjun D. Kumar, Li-Wei Chang, Matthew J. Ellis, Ron Bose

**Affiliations:** 1 Division of Oncology, Department of Medicine, Washington University School of Medicine, St. Louis, Missouri, United States of America; 2 Computational and Systems Biology Program, Division of Biology and Biomedical Sciences, Washington University in St. Louis, St. Louis, Missouri, United States of America; 3 Department of Pathology and Immunology, Washington University School of Medicine, St. Louis, Missouri, United States of America; 4 Siteman Cancer Center, Washington University School of Medicine, St. Louis, Missouri, United States of America; Duke-National University of Singapore Graduate Medical School, Singapore

## Abstract

A major goal of cancer genome sequencing is to identify mutations or other somatic alterations that can be targeted by selective and specific drugs. dGene is an annotation tool designed to rapidly identify genes belonging to one of ten druggable classes that are frequently targeted in cancer drug development. These classes were comprehensively populated by combining and manually curating data from multiple specialized and general databases. dGene was used by The Cancer Genome Atlas squamous cell lung cancer project, and here we further demonstrate its utility using recently released breast cancer genome sequencing data. dGene is designed to be usable by any cancer researcher without the need for support from a bioinformatics specialist. A full description of dGene and options for its implementation are provided here.

## Introduction

Cancer genome sequencing studies are now analyzing 50 to 500 patients per study and are documenting thousands of somatic mutations [1], [2]. New tools for annotation and analysis are needed to predict the functional relevance of these genetic alterations and guide subsequent investigations. Here, we introduce a tool based on druggable genes which, in combination with other annotation and filtering steps, can rapidly prioritize a large set of mutations into a more focused set that can be tested in functional studies.

This tool, which we call dGene (collection of Druggable Genes), is based on the concept of the druggable genome introduced by Hopkins and Groom in 2002 [3]. They identified protein classes that can potentially bind small molecule drugs and proposed that disease-modifying genes belonging to a druggable class should be prioritized for drug development [3], [4]. This set of druggable genes was based on the observation that FDA approved drugs and compounds in development do not target the human genome uniformly, with some gene classes, such as G-protein coupled receptors (GPCR) and protein kinases, being more frequently targeted by small molecules.

dGene adds to their work by expanding and updating the set of druggable classes based on current drug development efforts, populating classes comprehensively and maintaining quality through manual curation. In this article, we describe the rationale and construction of dGene, demonstrate its utility in a recently released set of breast cancer whole-genome and whole-exome sequence data [2] and provide instructions for using dGene.

## Results

**Figure 1:**
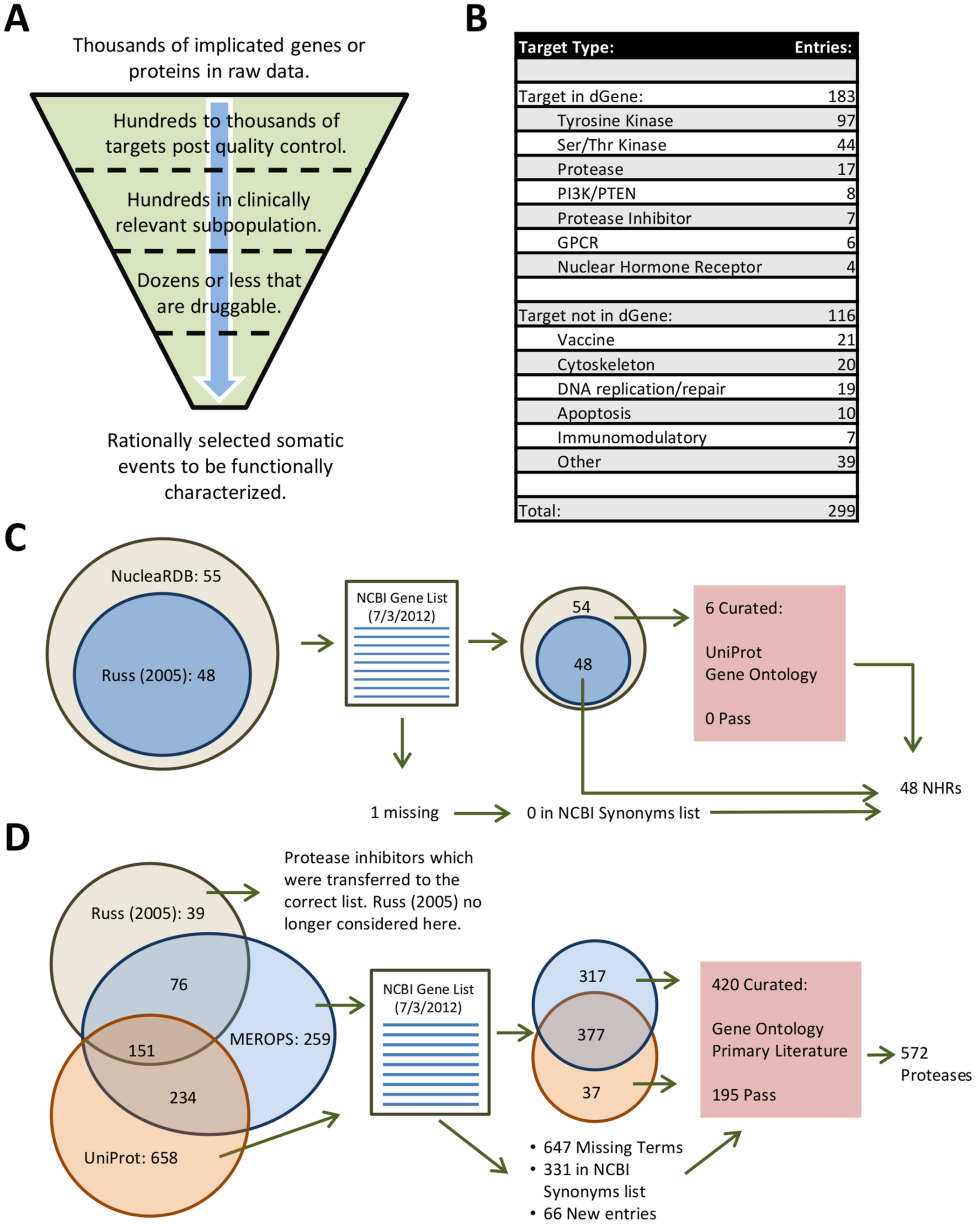
Rationale and process for construction of the dGene list. **A**, Druggability serves as a rational screen in a hypothetical pipeline for reducing a raw gene list to an experimentally workable number. **B**, Lung cancer drugs in the pipeline classified by target type, with some target types considered broadly druggable and included in dGene. **C**, NHRs required a simple workflow. Russ *et al,* 2005 and NucleaRDB [6] provided input. One gene mapped to neither the NCBI gene nor synonyms list. Six genes were identified in only one source and were manually checked against UniProt and Gene Ontology (GO) [9], [10]. None could be confirmed as NHRs, leaving the final class with 48 members. **D**, The elaborated workflow for proteases is analogous to that of the NHRs and other classes. Because UniProt served as input, curation involved searching the primary literature in addition to querying GO.

dGene is designed as an annotation and filtering tool for prioritizing mutations for functional assessment (Fig. 1a). The initial step in its design was selecting a set of gene classes that are both highly druggable and relevant to cancer biology. Classes were selected based on previous outlines of the druggable genome [3], [4] and additional probing of the primarily literature, with a particular emphasis on cancer biology. For instance, while transporters and ion channels are widely druggable, they have been excluded from dGene due to a lack of established relevance in tumorigenesis. The current version of dGene is built around ten gene classes (Table 1). We demonstrate the validity of this approach by examining a group of 299 drugs undergoing clinical trials for lung cancer [5]. We observed that over 60% of these drugs targeted proteins that are within the 10 classes in dGene (Fig. 1b).

**Table 1:** Summary of the dGene list.

Class	Description	Entries	Source(s)
**GPCR**	G-protein coupled receptors	857	Russ (2005); GPCRDB; UniProt
**PROTEASES**	Proteases	572	Russ (2005); MEROPS; UniProt; Gene Ontology
**ST_KINASE**	Serine/Threonine kinases	417	Russ (2005); Kinase.com; UniProt
**PROT_INHIB**	Protease inhibitors	153	Russ (2005); MEROPS; UniProt; Gene Ontology
**Y_KINASE**	Tyrosine kinases	91	Russ (2005); Human Kinsome; UniProt
**PTP**	Phosphotyrosine phosphatases	82	Russ (2005); Tonks (2006); Alonso (2003); UniProt
**NHR**	Nuclear hormone receptors	48	Russ (2005); NucleaRDB
**PTP_MTMR**	Myotubularin related phosphotyrosine phosphatases	16	Tonks (2006); Alonso (2003)
**PI3K**	Phosphatidylinositol 3 kinases	14	Engelman (2006); Gene Ontology
**PTEN**	Phosphatase and tensin homologues	7	Tonks (2006); UniProt; Gene Ontology
	Total:	2257	

The following references outline primary database construction: GPCRDB (Ref. 8; url: http://www.gpcr.org/7tm/); MEROPS (Ref. 7; url: http://merops.sanger.ac.uk/); KinBase (Ref. 11; url: kinase.com); NucleaRDB (Ref. 6; url: http://www.receptors.org/nucleardb/); Uniprot (Ref. 9; url: www.uniprot.org); Gene Ontology (Ref. 10; url: www.geneontology.org). All URLs valid as of 2/26/2013.

Each of the 10 dGene classes was comprehensively populated using tailored sources including specialized databases and review articles. For a given class, results from several sources were reconciled through the NCBI Gene List and entries unique to a single source were confirmed against databases like UniProt or the primary literature. Nuclear hormone receptors (NHR) illustrate a straightforward case with well curated sources [6] requiring little additional scrutiny (Fig. 1c). For comparison, proteases required an elaborated workflow involving additional specialized sources [7] and a greater degree of manual curation including primary literature searches (Fig. 1d). The final dGene list includes 2257 genes from the ten classes (Table 1 and Table S1), and draws from a variety of specialized and general sources [6]–[14]. dGene is entirely modular and expandable: future information or gene classes of interest can be easily added.

**Figure 2:**
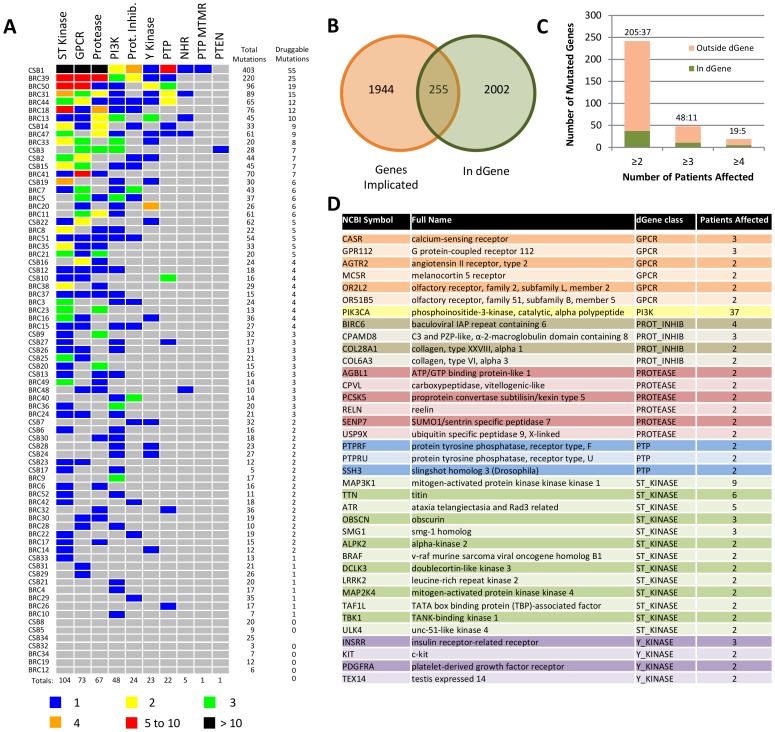
Applying the dGene list to SNVs in 77 breast cancer tumours. **A,** 368 SNVs occurred in genes considered to be druggable out of 2622 events total. **B,** 2199 genes had at least one SNV, of which 255 are considered druggable. **C,** Screening for commonly altered genes further reduces target list. **D,** 37 dGene entries present in at least 2 out of 77 samples, organized by class and patients affected.

The dGene filter has recently been used by The Cancer Genome Atlas (TCGA) Squamous Cell Lung Cancer project to analyze somatic mutations found in 178 squamous cell lung cancer cases; details can be found in that publication [1]. To further illustrate the utility of dGene, we chose a recent genomic study of 77 estrogen receptor positive breast cancers as a test case [2]. The dataset consists of 46 breast cancers that underwent whole genome sequencing, plus 31 cancers that underwent exome sequencing, denoted by “BRC” and “CSB” patient codes, respectively. dGene identified 368 single nucleotide variants (SNV) out of 2622 total as occurring in 255 druggable genes (Fig. 2a–b). Requiring recurrence in multiple patients reduces the gene set even further (Fig. 2c). The 37 genes which are both druggable and present in at least 2 patients are listed in Figure 2d. The input file and the dGene output file from this analysis are provided (Tables S2 and S3).

The dGene results provide new information about this cancer genome dataset. *PIK3CA* is mutated in 37/77 samples, but an additional patient (BRC44) had a KPDL567 in-frame deletion in PIK3R1, a regulatory subunit that binds PIK3CA. This deletion occurs at the PIK3R1-PIK3CA binding interface and may alter PI3-kinase signaling [15]. dGene suggests the importance of this mutation through both its relationship to PIK3CA and potential druggability. Additional mutations were similarly highlighted; for instance, the *TEX14* (names: testis-expressed protein 14 or sugen kinase 307) and *INSRR* (insulin receptor-related receptor) tyrosine kinases are two relatively novel drug targets. TEX14 has been implicated in multiple myeloma and breast cancer [16], [17], and INSRR has been implicated in ovarian epithelial cancers and neuroblastomas [18], [19]. Both are likely druggable, but neither occurred at high frequency and were not highlighted in a global analysis of the dataset. In order to demonstrate the value of the dGene results, comparison was made to search results from an existing drug database, the PharmGKB (The Pharmacogenomics Knowledgebase). dGene identified more genes than PharmGKB from this breast cancer dataset (Figure S1, Table S4), including identifying 4 tyrosine kinases and 13 S/T kinases that were recurrently mutated in these breast cancer genomes (Fig. 2D).

Figure 2d also illustrates two caveats in using dGene. Mutations in *MAP3K1* are found in 9/77 patients, and most of these events are loss of function mutations [2]. *MAP3K1*’s presence in the dGene output analysis demonstrates that dGene provides no information as to whether a mutation is gain-of-function, loss-of-function, or functionally silent. Given a list of gene symbols, dGene only acts as a filter. The presence of *Titin* and two collagen genes (*COL28A1* and *COL6A3*) illustrate how very large genes, which frequently contain druggable components and tend to be frequently mutated, will continue to filter through dGene. The presence of a gene in the dGene output does not guarantee a given mutation’s biological relevance.

**Figure 3:**
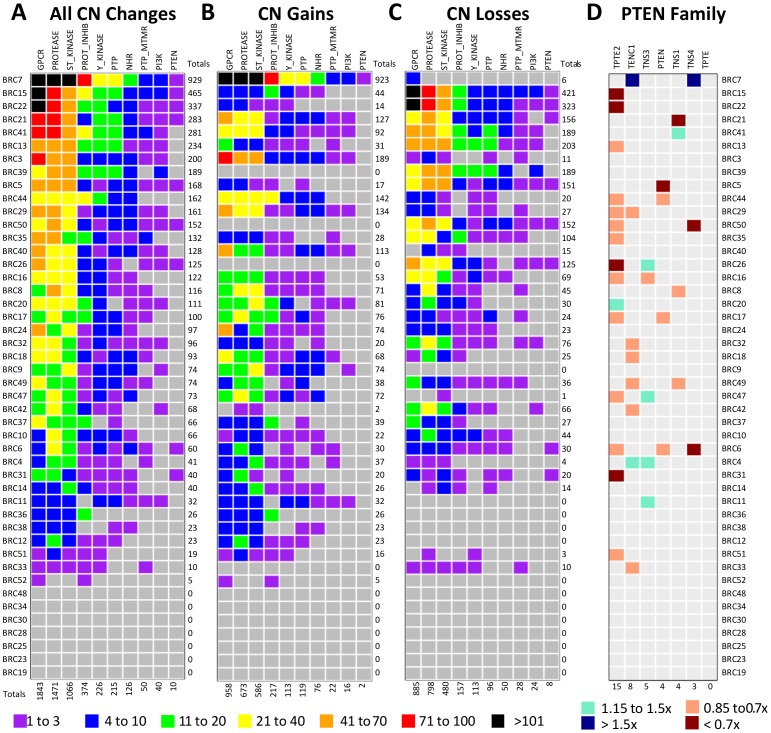
Applying the dGene list to CNVs in 46 breast cancer tumours. **A,** 5421 CNVs were detected in 1752 druggable genes across the sample. The 20^th^ (0.7×) and 80^th^ (1.5×) percentiles served as cutoffs. **B,** Gains only (>1.5×). **C,** Losses only (<0.7×). **D,** Displaying PTEN family CNV values. *TPTE2* is the most frequently altered. Cutoffs are relaxed to <0.85× and >1.15× for display purposes.

dGene can be applied to any dataset containing a list of gene symbols. To illustrate this we analyzed gene copy number (CN) data from the 46 estrogen receptor positive breast cancers that underwent whole genome sequencing (coded “BRC”) [2]. The raw CN data implicated 19,528 genes through nearly 150,000 events, including both focal and broad CN changes. As an initial screen, only events below the 20^th^ or above the 80^th^ percentile were considered (0.7× and 1.5× changes, respectively), leaving 54,301 events in 16,924 genes (Table S5). Filtering against dGene further reduced the set to 5421 CN changes in 1752 druggable genes (Figure 3a–c and Table S6). The CN losses in the PTEN family revealed a novel observation (Figure 3d). *TPTE2* (names: transmembrane phosphoinositide 3-phosphatase and tensin homolog 2 or TPIP) is the most commonly lost PTEN family member, with CN losses observed in 14/46 patients, which is a frequency 3.5-fold higher than the *PTEN* CN losses (4/46). The literature on TPTE2 is limited and it indicates that TPTE2 can inhibit cell growth and initiate apoptosis, similar to the PTEN tumor suppressor [20], [21], [22]. This novel finding of TPTE2 CN loss was identified because dGene highlights the association among PTEN family members from a large candidate CN alteration set.

## Discussion

We have developed an updated version of the druggable genome by identifying highly druggable gene classes, populating the classes using up-to-date and specific resources, and manually confirming the results. Our collection of druggable genes, dGene, is specifically tailored for use against mutation lists generated by cancer genome sequencing, though it can be used to analyze any human gene list. We have also shown that, in combination with additional filtering criteria, dGene can rapidly highlight mutations in biologically and clinically plausible therapeutic targets.

Limitations of dGene are that it is biased towards the “oncogene addiction” model of cancer and towards targets of well-described, small molecule drugs. While dGene does not currently contain genes involved in DNA repair, cell surface proteins, or other potential drug targets, additional classes are easily accommodated due to dGene’s modularity. dGene also makes no attempt to identify mutations as being either loss or gain of function; however, dGene can be combined with functional impact scores (such as Sift or Mutation Assessor) to identify mutations that are both likely druggable and likely functional [23], [24]. dGene is intended as a discovery phase tool to steer experiments towards genes against which small molecule inhibitors might quickly be developed.

As with all data-based resources, updating dGene will be of the utmost importance. dGene classes tend to be well studied, as illustrated by the fact that 2108 out of 2257 entries can be found in SwissProt, a manually reviewed collection of protein annotations [9]. Therefore, we anticipate dGene being quite stable, and are committed to providing annual updates. Moreover, because dGene is easily expandable, we can easily integrate new gene classes as knowledge of cancer biology advances and additional gene classes are targeted.

dGene is designed to be used by cancer researchers and not require support from a bioinformatics specialist. dGene is currently hosted as a web-based tool through the Genome Institute at Washington University (dgidb.genome.wustl.edu). There, users can filter gene lists against dGene (via the “Search Categories” page, or download the full dGene tab-delimited text file (via the “Downloads” page), which can be imported into various statistical packages and used or customized as needed. Additional functionality of the website includes annotating dGene entries with specific drug information where available (M. Griffith and O.L. Griffith, manuscript in preparation). In summary, dGene provides a rapid filter to identify druggable genes across ten classes from cancer genomic studies, and is currently available for use through a professionally constructed website.

## Methods

### Populating Gene Classes

Classes were populated with human genes through a process of inclusion from specialized databases and reviews, standardization to the NCBI gene list, and manual curation of genes occurring in a single source. Figure 1c and 1d portray the process fully for nuclear hormone receptors (a simple case) and proteases (a complex case), while Table 1 outlines the set of specialized sources used for each class. Reviews and databases were identified by literature search and may not be exhaustive. Manual curation of genes suggested by only one source ensured genes were properly classified. For classes where UniProt/Gene Ontology was not required as input sources, a simple check against the UniProt/GO classification was performed. In the cases where UniProt/GO were provided as input to the class (as was the case for proteases), inspection of the referenced literature and sequence alignment was performed.

During manual curation, bias was towards inclusion. Genes were left in their respective class if they either showed sequence homology to a known member, or if experimental evidence suggested they had the appropriate functionality. Pseudogenes and genes encoding nonfunctional products were included if they showed homology to an included class member.

A frequent challenge in consolidating disparate sources was the mixing of incompatible gene and protein identifiers. Mapping to the NCBI human Gene List (url: ftp://ftp.ncbi.nih.gov/gene/DATA/GENE_INFO/Mammalia/Homo_sapiens.gene_info.gz, accessed on July 3, 2012) facilitated comparisons between sources. The NCBI human gene list represents the total collection of human genes recognized in the NCBI data base as well as current annotations, and is updated on a daily basis. The NCBI gene list provides a standard format for all dGene entries –15 columns, including the NCBI geneID, official symbol, and crucially, a list of synonyms used in the literature. To each entry a 16^th^ column, class, has been appended. Mapping was accomplished by converting protein names to gene names with the David Gene ID Conversion Tool [25], and by searching the list of synonyms provided in the NCBI file for terms that do not appear as an official symbol.

### Application of dGene to 77 Breast Cancer Samples

The raw mutation annotations analyzed in this work utilized up-to-date gene ID numbers. Mutations within genes which also appear in dGene were filtered to a separate table, and the class term from dGene was appended as a new column. Aggregation to patient and class allowed for the production of Figure 2a. Aggregation to patient and gene was required for the production of Figure 2b–d. The raw CN data were analyzed in the same manner, with the results portrayed in Figure 3.

### Software

Analysis was performed in R 2.15.1 for Windows. Heatmaps were produced in R using the base package, while additional figures and tables were produced with Microsoft Excel and PowerPoint.

## Supporting Information

Figure S1 (PDF)

Table S1 (CSV)

Table S2 (XLS)

Table S3 (XLS)

Table S4 (XLS)

Table S5 (XLS)

Table S6 (XLS)
