# Supplementary material for: Prioritizing Potentially Druggable Mutations with dGene: An Annotation Tool for Cancer Genome Sequencing Data
Source: PLoS One. 2013 Jun 27;8(6):e67980. doi: 10.1371/journal.pone.0067980 (PMC3694871; doi:10.1371/journal.pone.0067980)
Supplement: Figure S1 — (PDF) [file pone.0067980.s001.pdf]

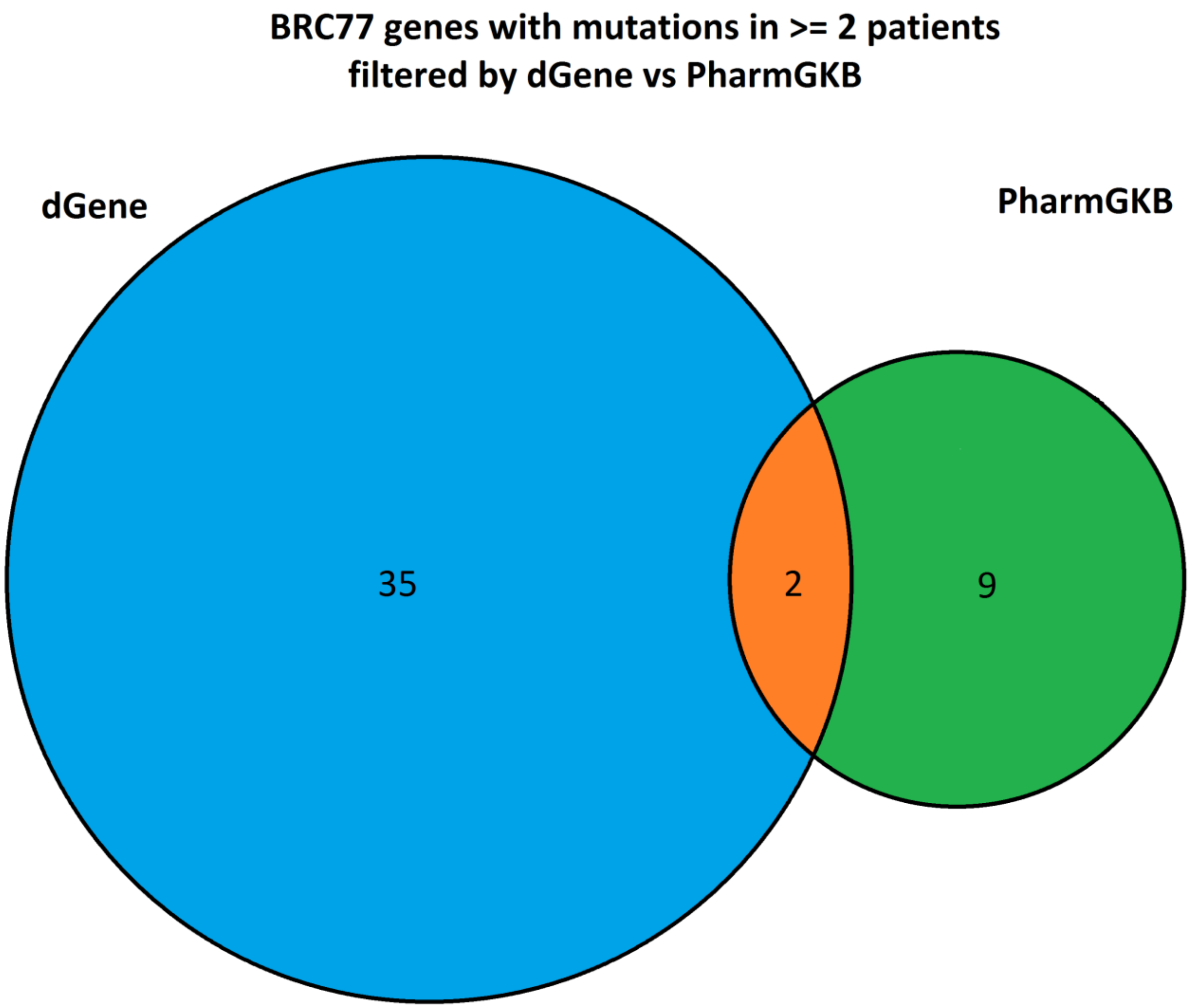

Supplementary Figure 1. 242 genes which were recurrently mutated in 2 or more patients in the BRC77 dataset were filtered against dGene and PharmGKB. The 37 entries found within dGene can be found in Figure 2D, while the 11 from PharmGKB are in Supplementary Table 4.
